# Basil Seeds as a Novel Food, Source of Nutrients and Functional Ingredients with Beneficial Properties: A Review

**DOI:** 10.3390/foods10071467

**Published:** 2021-06-24

**Authors:** Héctor Calderón Bravo, Natalia Vera Céspedes, Liliana Zura-Bravo, Loreto A. Muñoz

**Affiliations:** 1 Food Science Lab, School of Engineering, Universidad Central de Chile, Santiago 8330601, Chile; hector.calbravo@gmail.com (H.C.B.); nati.vcespedes@gmail.com (N.V.C.); liliana.zura@gmail.com (L.Z.-B.); 2 Department of Food Science and Chemical Technology, Universidad de Chile, Santiago 8380494, Chile

**Keywords:** basil seed, functional ingredients, *Ocimum basilicum* L., oilseed, novel food

## Abstract

Basil (*Ocimum basilicum* L.) is found worldwide and is used in the food, pharmaceutical, and cosmetic industries; however, the nutritional and functional properties of the seeds are scarcely known. Basil seeds contain high concentrations of proteins (11.4–22.5 g/100 g), with all the essential amino acids except S-containing types and tryptophan; dietary fiber (soluble and insoluble) ranging from 7.11 to 26.2 g/100 g lipids, with linoleic (12–85.6 g/100 g) and linolenic fatty acids (0.3–75 g/100 g) comprising the highest proportions; minerals, such as calcium, potassium, and magnesium, in high amounts; and phenolic compounds, such as orientine, vicentine, and rosmarinic acid. In addition, their consumption is associated with several health benefits, such as the prevention of type-2 diabetes, cardio-protection, antioxidant and antimicrobial effects, and anti-inflammatory, antiulcer, anticoagulant, and anti-depressant properties, among others. The focus of this systematic review was to study the current state of knowledge and explore the enormous potential of basil seeds as a functional food and source of functional ingredients to be incorporated into foods.

## 1. Introduction

*Ocimum basilicum* L., commonly known as basil or sweet basil, is an annual spicy herb of the Labiatae family. The name basil is derived from the Greek word “Basileus” meaning “Royal” or “King” and it is often called “King of the herbs” due to its wide range of uses in medicine, cosmetics, and the pharmaceutical and food industries [1].

This plant is originally from warm and tropical areas, such as India, Africa, and Southern Asia [2] and is specifically found in Pakistan and India, where it has been cultivated for around 5000 years. Today, it is found all over the world [3]. *O. basilicum* is commercially cultivated in many warm and temperate countries, including France, Hungary, Greece, and other southern European countries, as well in North and South America [4].

This herb has been used in different ways from ancient times; the leaves can be used fresh or dried to add a distinctive flavor and aroma to foods. It is also used in the manufacture of beverages, liqueurs, vinegars, drinks, teas, and cheese, among others and the essential oils, which are extracted from the leaves and flowers, are used in the food, pharmaceutical, and cosmetic industries [3,5]. The seeds are commonly added to beverages and ice cream and are also added whole or milled to bakery products as a source of dietary fiber for technological purposes.

Moreover, the seeds are used to enrich fruit-based beverages for visual and functional purposes [6,7,8]. The seeds are high in dietary fiber and, thus, have huge potential as a functional ingredient. The mucilage extracted from basil seeds has been widely studied, and has emulsifying, foaming, thickening, stabilizing, viscosity, and gelling properties, among others [8,9,10,11,12]. Basil seeds are not conventionally used as a food, despite the literature demonstrating that its consumption stands out not only for its nutritious value but also for its significant health benefits, such as antidiabetic, antimicrobial, antioxidant, and anticancer activities [13,14].

Finally, the branches and soft woody stem can be added as a flavoring agent to different foods, the flowers are used in different dishes and beverages [3], and the roots are traditionally used in Indian medicine [15]. In general terms, basil leaves, flowers, seeds, branches, soft woody, and roots are used in both domestic and industrial applications, such as the food, pharmaceutical, and cosmetic industries.

Basil has also been widely used in traditional medicine in the treatment of headaches, coughs, constipation, diarrhea, warts, worms, and kidney problems [16]. In addition, various pharmacological actions have been described, such as stomachic, antioxidant, antiviral, antimicrobial, analgesic, anti-inflammatory, antidiabetic, and anti-stress activities, and antipyretic diuretic and emmenagogue properties, among others [1,3].

The objective of this work is to present a systematic review of the current state of knowledge on basil seeds and their by-products from a food science point of view. We specifically highlight their nutritional, physical, chemical, and agronomic characteristics as a potential functional food, including the most recent research reported in literature.

## 2. Methods

A systematic review was conducted by searching electronic databases, including 102 articles. Relevant articles were selected on the basis of the nutritional, chemical, agronomical, and functional properties of basil seeds. The databases used were the Web of Science (https://clarivate.com/webofsciencegroup/solutions/web-of-science/), EBSCO (www.ebsco.com), and Scopus (www.scopus.com), among others.

## 3. Botanical and Agronomical Diversity of Basil

The genus *Ocimum* belongs to the *Lamiaceaea* family, which comprises more than 160 species distributed around the world [17]. This herbaceous plant is an erect, strongly aromatic, glabrous, branched herb that grows between 30 and 90 cm high [18]. The leaves are of ovoid shape, the color ranges from bright green to purple, and the flowers are white or pale purple and are arranged in long terminal racemose inflorescences [4].

Basil can tolerate different climatic and ecological conditions and grows from cool moist zones to tropical rain forest zones at temperatures between 6 and 24 °C; however, it favors warm climatic conditions [19]. The geographical distribution shows three main centers of diversity: the tropical and subtropical regions of Africa, tropical Asia, and tropical parts of Latin America (Brazil). The maximum number of species is found in the tropical rain forests of Africa [20,21].

The botanical classification of basil, as described by the USDA [22], is as follows:

- Kingdom: *Plantae*—plants
- Sub-kingdom: *Tracheobionta*—vascular plants
- Superdivision: *Spermatophyta*—seed plants
- Division: *Magnoliophyta*—flowering plants
- Class: *Magnoliopsida*—dicotyledons
- Sub-class: *Ateridae*
- Order: *Lamiales*
- Family: *Lamiaceae*—mint family
- Genus: *Ocimum* L.—basil
- Species: *basilicum*
- Binomial name: *Ocimum basilicum*—sweet basil

From the world market point of view, the most commercially important cultivars belong to the species *O. basilicum.* They are characterized by different morphological features, such as size, shape, color, and aroma. They also have diverse growth habits and types of leaves, flowers, steam colors, and chemical composition [23]. 

According to Darrah, Helen [24,25], *O. basilicum* cultivars can be classified into seven types: (i) tall slender types (the sweet basil group is commonly the green type with white flowers); (ii) the large-leaf robust type (lettuce leaf, also called Italian basil, with a less pronounced flavor); (iii) dwarf types, which are short and small leafed (bush basil, with small and pungent leaves); (iv) compact types, also described as *O. basilicum* var. *thysiflora* (Thai basil, characterized by a balm-like flavor); (v) purpurascens, the purple colored basil types, with a traditional sweet basil flavor; (vi) purple types (dark opal, an hybrid between *O. basilicum* and *O. forskolei* with a sweet basil plus clove-like aroma); and (vii) *citriodorum* types (lemon and lime-flavored basils). 

In addition to the traditional types of basil, other species have been introduced for culinary and ornamental purposes and potential sources of new aromas [23,24,25]. Moreover, certain varieties have been development to produce high yields with chemical variability: for example, CIM-Saumya, is a short duration crop and has a potential essential oil production of 85–100 kg/ha; CIM-Snigda was developed with a distinct leaf morphology and has a unique aroma; and CIM-Surabhi was developed as a high oil-yielding (100–120 kg/ha of essential oil) plant with a unique chemical composition [26].

## 4. Morphological and Physical Characterization of Basil Seed

The morphological and physical characterization of seeds is important due to the relationship between the shape and size of the seeds and the design of tools for crop production, storage facilities, and potential food application. Despite basil being an important commercial plant, there is a lack of data describing the seed morphology and physical characteristics [27].

Several authors described basil seeds as oval, ellipsoid, and small, with dimensions ranging from 2.31 to 3.11 mm in length, 1.3 to 1.82 mm in width, and 0.99 to 1.34 mm in thickness, as can be seen in Table 1. Their surface was described by Uematsu et al. [28] as porous from results obtained using Scanning Electron Microscopy (SEM) in Thailand basil seeds.

Basil seeds vary in size depending on the area in which they are planted and the country they are from. Kišgeci et al. [29] reported that seeds from the same country (Serbia), but collected from different localities, demonstrated significant differences in terms of length, width, and thickness. On the other hand, in the same work, Iranian basil seeds were shown to be bigger than Serbian basil seeds. These differences in size were correlated with moisture, i.e., the size increased as the moisture content increased [30]. Furthermore, the sizes of Iranian basil seeds were studied by Hosseini-Parvar et al. [31] and Razavi et al. [32]. They reported that, two seeds of a similar size demonstrated moisture contents of 9.1% and 5.5%, respectively.

**Figure 1:**
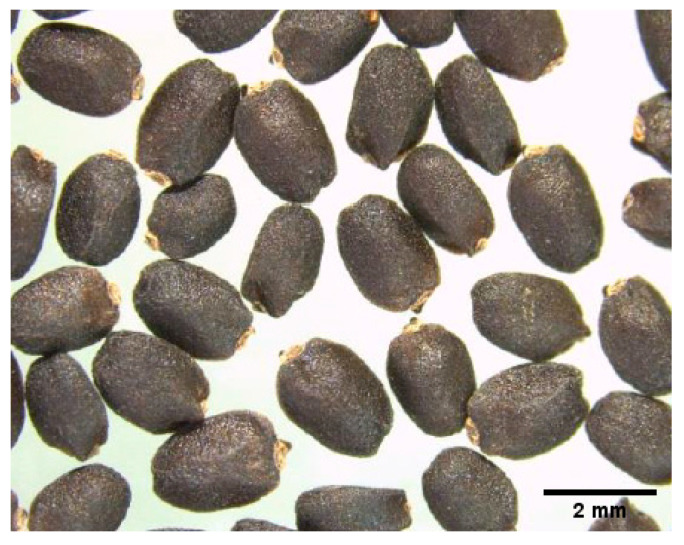
Basil seeds.

In the Figure 1, the image of basil seeds shows black coloring and porous surfaces. These characteristics were previously described by several authors [29,30,31,33]. Choi et al. [30] studied basil seed color in order to discriminate between types and concluded that the Singaporean basil seed color can be identified by the naked eye, but seeds from India, Pakistan, and Vietnam cannot be differentiated. The seeds from the same study had Cielab* values ranging between 19.62 and 26.28 to L*; 1.1 and 3.9 to a* and 3.57 and 5.78 to b* with significant differences in the seeds from Singapore.

Considering that seeds from different geographic locations have different characteristics, it would be interesting in future studies to include seeds from Latin and North American countries, mainly due to the differences in environmental conditions.

## 5. Biochemical and Nutritional Composition of Basil Seed

The consumption of basil seeds is not very common; however, in some Middle Eastern countries, they are used in foods and beverages. The consumption of this seed has not spread to the rest of the world mainly because its valuable nutritional and functional properties are unknown. Various studies have reported the nutritional composition of basil seeds, highlighting the biological value of seeds from different countries. This is shown in Table 2. In terms of energy, Khaliq et al. [5] performed calculations based on the percentage values of carbohydrates, proteins, and fats, and obtained an average value of 442.4 kcal. Moreover, the moisture content of the seeds ranged from 4.0 to 9.6 g/100 g. This variability can be attributed to the moment of harvest, climate and storage conditions [5]. 

Finally, basil seeds and other oil seeds, such as chia seeds, can vary in nutritional composition and bioactive compounds according to the agronomic management, environmental conditions, geographical location, altitude, soil properties, origin of the seeds, and degree of absorption of water, among other influences [8,30,34].

### 5.1. Carbohydrates

Carbohydrates are the principal source of energy in human metabolism [5]. This nutrient has complex chemical structures and performs a rich physiological function in living systems. Certain carbohydrates can play important roles in regulating the intestinal microbiota through prebiotic effects. These effects include the protection of the intestinal epithelial barrier, the suppression of inflammatory responses, decreasing lipogenesis, and elevating satiety hormone levels [30]. The benefits of basil seeds are mainly associated with their nutritional composition (Table 2), as they are a good source of carbohydrates. The high carbohydrates content, ranging between 43.9 and 63.8 g/100 g of seed (Table 2), not only represents the sugar content, but also the high content of dietary fiber.

The carbohydrate profile of basil seeds was first reported by Mathews et al. [10], and the results indicate that the seeds contained non-starchy polysaccharides in the form of cellulose (8.03%), hemicellulose (9.87%), and lignin (35.2%), with the highest proportion. In addition, in the same study, the seeds exhibited a high fiber content and were suggested as an unconventional source of dietary fiber. In this context, Rezapour et al. [6] used basil seed powder as a source of dietary fiber and other components to enhance the dough properties and improve the nutritional profile of baguette bread.

Many plants can produce complex polysaccharides, commercially known as plant-based gums. Plant gums exudate and seed gums are complex polysaccharides/carbohydrate polymers that are generally used as dietary fiber, fat replacers, thickening agents, foaming agents, films, emulsifiers, and stabilizers, for controlling ice crystal growth and drug delivery agents [37,38].

The hydrocolloids from seeds can be used in food formulations due to their affordable price, availability, and functionality [37,39,40]. In this context, the content of mucilage from basil seeds is about 17–20%, with functional properties comparable to those of various other commercial food hydrocolloids [27,31,37]. The potential of basil seed gum as a new source of hydrocolloid was investigated by Kim et al. [11] and Hosseini-Parvar et al. [41] as a fat substitute and stabilizer with excellent results.

Finally, *O. basilicum* seed gum is also used for many other purposes, such as a source of fiber, a disintegrant, a pharmaceutical excipient, a suspending agent, an anti-diabetic agent, for seedling growth, and a biodegradable edible film [42].

### 5.2. Proteins

Previous studies reported that protein deficiency is the most common type of malnutrition, and, depending on the duration and intensity, it can have multiple physiological consequences [43]. Plant-based foods that provide more than 12% of their calorific value in protein are considered to be remarkable suppliers of protein, which is particularly relevant today as interest in vegetarian and vegan diets is increasing. The data presented in Table 2 indicates that sweet basil seeds have high protein contents, ranging between 10% and 22.5%. These findings suggested that basil seeds are a good source of proteins, which is valuable for human health from a nutritional point of view [44].

In addition, the amino acid composition of basil seeds illustrates the high nutritional quality of the protein (Table 3). In this context, only one report by Karnchanatat et al. [45] was found on the amino acid composition of *Ocimum basilicum*, the cultivar of hoary basil seeds, which was compared with *Ocimum tenuiflorum* seeds in a study developed by Ziemichód et al. [46]. The results showed that glutamic acid and aspartic acid were the major non-essential amino acids in *O. basilicum* seeds. Furthermore, all essential amino acids, except S-containing types and tryptophan, are present in high amounts in this species, which make it very attractive from a nutritional point of view in terms of dietary intake recommendations [44].

### 5.3. Lipids

According to the data in Table 4, basil seeds have a fat content ranging between 9.7% and 33.0% indicating that the seeds are a good source of lipids. The differences observed in seeds from different countries can be attributed to genetic and environmental factors, such as temperature and precipitation, the efficiency and parameters used during extraction, including solvent type, temperature, extraction time, and the size of the seeds and their moisture contents [47,48,49,50]. In addition, according to Nazir et al. [35] high lipid contents and low protein contents can be explained by variations in the altitude of the ecosystem in which the seed is grown.

Lipids are stored in high concentrations in different plant seeds, presumably because lipids contain approximately twice the amount of energy per unit dry mass as compared to carbohydrates [50].

Moreover, fatty acids are the main nutritional components in edible oilseeds, and a growing body of evidence suggests that individual fatty acids may provide human health benefits. The incorporation of polyunsaturated (n-3) fatty acids, and essential fatty acids, such as linoleic (LA), linolenic (ALA), and arachidic fatty acids, in the diet can play a natural preventive role in cardiovascular disease and other health problems and diseases [42,54,55]. In this context, basil seeds are a good source of polyunsaturated fatty acids. Table 4 shows the predominant fatty acids in the seed oil according to the literature. The main unsaturated fatty acids were ALA (0.3–66.0%) and LA (12–85.6%), followed by oleic acid (8.5–13.3%). The most abundant saturated acids included palmitic acid (4.9–11.0%) and stearic acid (2.0–6.6%).

In Table 4, seeds from the different studies and countries show differences in the composition and contents of fatty acids: for example, according to Choi et al. [30], basil seeds from Singapore exhibited a higher ALA content and a lower content of LA compared with the samples from Indian, Pakistan, and Vietnam. This can be explained by the inversion of LA and ALA in basil seed oil that occurs in certain species [51]. Furthermore, in Table 4, the samples from Iran [47], Canada [51], and Vietnam [30] exhibited higher amounts of ALA, while the Singapore [30], Sudan [42], and United Arab Emirate [52] samples had higher amounts of LA. These differences in the fatty acid compositions can be attributed to environmental and climatic factors; although, according to Mostafavi et al. [33] and Choi et al. [30], the plant genotype is the most important parameter.

Since basil oil has a high amount of ALA (C18:3), it could be a promising source of omega-3 for vegetarians and vegans. Finally, according to Mostafavi et al. [33], the fatty acid content not only determines the nutritional, medicinal, and industrial properties of herbs, it also affects plant responses to stress.

### 5.4. Minerals

Minerals are considered inorganic components of plant materials and are important nutritionally [5,56]. In addition, their incorporation into the diet plays an important role in the management of diseases and wellbeing, despite the fact that they comprise only 4% to 6% of the human body [56,57]. The principal minerals that are required in higher amounts include calcium, phosphorus, magnesium, sulfur, potassium, chloride, and sodium, which are classified as macronutrients, are structural components of tissues, and function in the cellular and basal metabolism, and water and acid-base balance. Trace minerals, which are considered as micronutrients, include zinc, iron, silicon, manganese, copper, fluoride, iodine, and chromium and are very important for hormones, vitamins, and enzyme activity [57,58]. An insufficient supply of mineral elements in the diet can have negative effects, such as causing learning disabilities in children, increasing morbidity and mortality, reducing worker productivity, and increasing healthcare costs [59].

As is the case with amino acid composition, there are only a few studies concerning the mineral composition of basil seeds in the literature. Table 5 shows a comparison of the mineral compositions of *O. bacilicum* and *O. tenuiflorum* seeds according to studies by Munir et al. [34] and Ziemichód et al. [46], respectively.

The results show that, according to the Dietary Reference Intake (DRI) values, basil seeds are a good source of minerals [44]. In this context, calcium and potassium were found in high amounts in *O. tenuiflorum* (636 and 481 mg/100 g, respectively), followed by magnesium, with values of 31.55 and 293 mg/100 g for *O. basilicum* and *O. tenuiflorum* respectively, and iron, zinc, sodium, and manganese in minor proportions. Elements, such as phosphorous, potassium, calcium, magnesium, iron, zinc, copper, and manganese, are the most important minerals for the human body and play important roles in disease development and prevention [59]. 

Calcium is generally known for its role in regulating muscle contraction and maintaining skeletal integrity, while magnesium is involved in several functions, including signaling pathways, energy storage and transfer, glucose metabolism, lipid metabolism, neuromuscular function, and bone development [60,61,62]. Moreover, potassium plays a critical role in normal cellular function and participates in carbohydrate metabolism and protein synthesis [63]. According to the Food and Nutrition Board [44], the daily requirements of calcium, magnesium, and potassium for an adult are 310–400, 1000, and 2600–3400 mg/day, respectively. To this end, basil seeds can supply 100% of the Ca, around 50% of Mg, and around 20% of K according to the requirements.

In general terms, the seeds of *O. basilicum* are characterized as having a lower mineral content as compared to *O. tenuiflorum* seeds. These differences may be attributed to various elements, such as growth conditions, genetics factors, geographic variations, and analytical procedures [64,65].

## 6. Beneficial Properties of Basil Seeds

### 6.1. Antioxidant Activity

It is well known that phenol compounds perform various physiological functions in plants and their intake produces protective effects against certain serious diseases, such as cancer and cardiovascular disease [66]. In the case of basil, the antioxidant activity of the plant has been widely studied; however, the seeds have scarcely been analyzed.

In general, the literature agrees that basil seeds have good antioxidant potential, even better than other seeds, such as sesame or red seeds, and could be used to develop new natural antioxidants or be included as ingredients to prevent oxidative deterioration in foods [36,67,68]. In particular, the antioxidant capacity (AOA) and total phenolic content (TPC) of basil seeds were determined by various research groups using the DPPH (2,2-diphenyl-1-picryl-hydrazyl-hydrate) and Folin–Ciocalteu methods, respectively, each reporting different values (Table 6).

The results by Mezeyová et al. [69] are not comparable with others, as the calculation formula was different. However, seeds from Pakistan presented lower values of TPC (4890 µg GA/g), and seeds from Iran demonstrated higher values (22,900–65,500 µg GA/g).

Other factors that can influence the results are differences in the initial DPPH concentrations, reaction time, and type of solvent used to prepare the extract, as reported by Safraz et al. [36]. Table 6 shows that, when comparing the results from different studies, the AOA values are higher when using methanol as the extraction solvent, obtaining values between 34.2 and 968.49% AOA; this is followed by petroleum ether with an AOA of 73.85%; and *n*-hexane with 57.35%. According to Safraz et al. [36], these differences can be attributed to the presence of more polar than nonpolar compounds, which means higher yields are obtained with methanol than with n-hexane. 

Although it is difficult to fully characterize basil seed extracts, it is possible to determine that the antioxidant capacity is mainly provided by phenolic compounds, followed by other secondary antioxidant metabolites, such as carotenoids, volatile oils, and others [13]. According to Javanmardi et al. [70] and Cherian [71], in terms of flavonoids and phenolic acid contents, the amounts of orientine, vicentine, and rosmarinic acids are remarkable because they are the most abundant phenolic compounds in *Ocimum* spp. In addition, in a recent study by Ghaleshahi et al. [47], interesting findings were reported concerning tocopherol, i.e., basil seeds contained significantly higher concentrations of α, β, and γ-tocopherol when compared with flax and perilla seeds. 

In the same study, basil seeds were shown to contain higher amounts of sterols over flax and perilla seeds, and surprisingly, a higher amount of phytosterol was found, compared with in extra virgin olive oil and safflower. Moreover, Mabood et al. [67] reported that basil seeds contained higher amounts of TPC as compared with Sesame seeds, Ajwan seeds, and Red seeds. In this context, in a study by Gajendiran et al. [13], the presence of different phytochemicals, such as saponins, terpenoids, flavonoids, tannins, steroids, and alkaloids, was also revealed. Finally, a recent study by Afifah and Gan [72] found that basil seeds also contained bioactive peptides with antioxidant properties.

### 6.2. Antimicrobial Activity

In recent years, various pathogens have demonstrated a resistance to drugs. This has led to a search for new naturally derived antimicrobial agents, and researchers have, accordingly, begun to pay special attention to plants and their seeds, including sesame, soybean, chia, and basil seeds.

In particular, several authors reported the antimicrobial effects of basil seed oil against Gram-positive and Gram-negative bacteria. In this context, Gajendiran et al. [13] demonstrated its effectiveness against nine clinical pathogens (*Staphylococcus aureus*, *Escherichia coli*, *Enterococcus* spp., *Proteus mirabilis*, *Shigella dysenteriae*, *Salmonella* spp., *Klebsiella pneumoniae*, *Serratia marcescens*, and *Pseudomonas aeruginosa*), showing that it was most effective against *Pseudomonas aeruginosa* at a concentration of 100 mg oil/mL. In addition, in a study by Singh et al. [73], the oil from *Ocimum sanctum* seeds showed good antibacterial activity against various pathogens. 

They reported that *Staphylococcus aureus* was the most affected organism as compared with *Bacillus pumius* and *Pseudomonas aeruginosa*; lower levels of activity were reported against *Escherichia coli*, *Klebsiella pneumoniae*, *Salmonella typhi*, and *Staphylococcus epidermidis*; and it was shown to be inactive against *Bacillus subtilis* and *Micrococcus luteus*. It was also determined that the antibacterial effect of these fatty acids could be mainly related to their degree of unsaturation; thus, linolenic fatty acid would be the fatty acid that contributes the most to antibacterial activity.

In addition, Majdinasab et al. [74] studied the antimicrobial activity of coatings based on the mucilage of basil seeds, due to the protection endowed by the coating against oxygen and agents that affect food. This antimicrobial action could be enhanced by combining this coating with an essential oil, such as *Shizari thyme* essential oil, in order to increase the quality and shelf life of meat products.

### 6.3. Benefits of Fatty Acids from Basil Seeds

Fixed oils are glycerol esters of varying consistencies that are found in both animals and plants. The ω6 (n6) series derived from linoleic acid (18:2, n-6) and the ω3 (n3) series derived from α-linolenic acid (18:3, n-3) are groups of essential fatty acids for the body. These acids provide energy, are an integral part of cell membranes, and are precursors of eicosanoids (prostaglandins, thromboxanes, and leukotrienes). Eicosanoids participate in the development and synthesis of immune and inflammatory responses [75]. Numerous properties of basil seed fixed oils are reported in the literature as detailed below.

The anti-inflammatory capacity was reported by Singh et al., 2008 [75], in seeds containing α-linolenic fatty acid (ALA). In this study, 1.0, 2.0, and 3.0 mL/kg doses of basil seed, linseed, and soyabean fixed oils were used for the analysis. Each dose, containing ALA, was used in models of carrageenan, leukotriene, and arachidonic acid-induced paw edema in rats. The result showed that higher inhibition was produced by oils with higher ALA contents from basil seeds and linseeds in leukotriene-induced paw edema. This behavior suggests that modulation of the course of inflammatory disorders can be achieved by a dietary intervention, i.e., modifying the availability of polyunsaturated fatty acids.

In a study carried out by Singh and Agrawal [76], the anti-asthmatic and the anti-inflammatory activities of the fixed oil extracted from basil seeds were evaluated in guinea pigs. The results showed that the fixed oils from basil seeds significantly protected against histamine and acetylcholine-induced models. Moreover, anti-inflammatory activity against carrageen-induced paw edema in rats was also confirmed.

In another study developed by Singh and Majumdar [77], the antipyretic activity of the fixed oil of basil seeds (*O. sanctum*) was evaluated. It was tested against typhoid-paratyphoid fever A/B vaccine-induced pyrexia in rats. They observed that, at doses of 1.0 mL/kg or higher, the oil exhibited a defined antipyretic property. Moreover, the analgesic activity of the fixed oil of basil seeds was studied [78]. This was carried out by intraperitoneal injection of mice and rats at doses of 1.0, 2.0, and 3.0 mL/kg of the oils. The results showed that the oil produced significant inhibition in a dose-dependent manner, suggesting a possible peripheral system-related mechanism.

In another study, the effect of the fixed oil of basil seeds on arthritis was evaluated [79]. In this work, arthritis was induced in two ways: by injecting a *Mycobacterium tuberculosis* suspension and by injecting a formaldehyde solution into rats. As a result, it was determined that the fixed oil of basil seeds significantly inhibited paw edema and significantly decreased inflammation and arthritic nodules at a dose of 3.0 mL/kg.

Additionally, the antiulcer activity of the fixed oil of basil seeds against aspirin-, indomethacin-, alcohol-, histamine-, reserpine-, serotonin-, and stress-induced ulceration in rats and guinea pigs was evaluated [80]. These authors used oil doses of 1.0, 2.0, and 3.0 mL/kg, noting a significant reduction in the antiulcer effects in experimental animal models.

The antihyperlipidemic and antioxidant effects of basil seed oil were also investigated in rabbits [81]. The results showed that the dietary supplementation of *O. sanctum* seed oil for four weeks significantly reduced the serum cholesterol triacylglycerol and LDL-cholesterol + VLDL-cholesterol (LDL: Low-density lipoprotein; VLDL: Very low-density lipoprotein). In addition, this supplementation also decreased lipid peroxidation and reduced the glutathione (GSH) levels in the blood. Therefore, this study confirmed the cholesterol-lowering and antioxidant effects of this oil.

In order to determine the anticoagulant and hypotensive effect of the fixed oil of basil seeds, doses of 3.0 mL/kg were applied intraperitoneally to rats [82]. An increase in the blood clotting time was observed. This increase was comparable to aspirin, which may be due to an antiaggregant action on platelets. With these results, it was possible to verify the anticoagulant capacity of the fixed oil.

In addition, the chemo-preventive activity of basil seed oil against induced fibrosarcoma tumors was evaluated [83]. A maximum oil dose of 100 µL/kg of body weight was supplied, producing a significant reduction in the induced tumor incidence and tumor volume. Other biological activities of certain extracted seed oils, such as antioxidant, antimicrobial, anticancer, and anticoagulation activities, have been previously described in the literature [84,85,86].

Finally, in a recent study by Idris et al. [42], the physicochemical characteristics and fatty acid composition of *O. basilicum* seed oil were reported. It was shown that this oil can be used in countless applications due to the high content of essential fatty acid, such as LA and ALA. They also suggested that *O. basilicum* oil could have applications in the paint, varnish, ink, and cosmetic industries, in addition to alternative uses in industries in which the use of these fatty acids is required.

A summary of the biological activity of basil seeds and their constituents is presented in Table 7.

### 6.4. Uses of Basil Seeds in Traditional Medicine

Basil seeds are traditionally used as a natural remedy for the treatment of indigestion, ulcers, diarrhea, sore throats, and kidney disorders [1,31,67,87]. Basil seeds have also been used as a diuretic, antipyretic, aphrodisiac, and anti-dysenteric [1,67]. Traditionally, the consumption of basil seeds soaked in water provides a refreshing and nourishing food. The seeds, washed and pounded, are used in poultices for sores and sinus problems and are also used for the treatment of chronic constipation and internal piles [4]. The seeds are chewed as an antidote to snake bites [1,23]. 

The daily consumption of an infusion prepared with a teaspoon of seeds in a glass of water and sugar acts as a demulcent in the treatment of genitourinary disorders. An infusion of seeds relieves pain after childbirth and has also been given to reduce fever [88]. In addition, basil extracts have a number of useful properties, including bactericidal, anti-inflammatory, antioxidative, antiulcer, antidiarrheal, and chemo-preventive effects. They lower blood sugar, stimulate the nervous system, protect against radiation, and protect against oxidative DNA damage and mutagenesis [67].

### 6.5. Other Benefits

A study by Gajendiran et al. [13] revealed the presence of different phytochemical constituents, such as alkaloids, flavonoids, carbohydrates, tannins, and terpenoids in extracts of petroleum ether from *O. basilicum* seeds. In this study, the seeds were shown to have good antimicrobial, antioxidant, and anticancer activities.

Imam et al. [14] studied the antidiabetic activity of water-soluble polysaccharides from *O. basilicum* seeds by measuring the inhibitory activity for protein tyrosine phosphatase 1B in vitro. In addition, Afifah and Gan [72] found that basil seeds contained peptides with an antioxidant activity, as previously mentioned. Moreover, α-amylase inhibitory activities and three novel inhibitor peptides were successfully identified. It was suggested that these peptides can be used as therapeutic agents for reducing the risk of oxidative stress and to prevent type-2 diabetes.

The selenium accumulating properties of basil seeds have been used to produce selenium-biofortified microgreens in an attempt to increase the content of this mineral and, thus, its intake by humans [89].

## 7. Uses of Basil Seeds and By-Products

### 7.1. Food Uses

Basil seeds are used in different products for culinary, nutritional, pharmacological, and aesthetic purposes, and are common in many Asian countries, such as Iran and India. In these countries, the seeds are consumed frequently in drinks (Sharbat) and frozen desserts (Faloodeh) for aesthetic purposes and as a source of dietary fiber [10,31,34,71]. A study by Munir et al. [34] showed that a drink with up to 0.3% basil seeds had good sensory properties, such as taste, texture, and acceptability; moreover, there was an increase in the fiber and protein contents, and provided a significant amount of minerals and phenolic compounds, as compared to the control drink.

Several research groups investigated the application of mucilage from basil seeds in different food products due to its technological, functional, and nutritional properties. The mucilage from basil seeds has various uses, e.g., as a water binding agent in low-salt meat product [90]; as a fat substitute in sponge cakes, reducing fat content by 75% [91]; as a gelling and stabilizing agent in pudding (milk protein gel), ice cream, and low fat yogurt due to its interaction with milk protein. This was shown to improve their rheological properties, decrease syneresis, and provide high gel strength [11,92,93]; and as an additive to improve the physicochemical and sensory properties of bread and other bakery products [94].

Despite basil seed oil demonstrating useful properties for industrial purposes due to its oil content and composition and being processed in the same way as linseed oil [51], it has yet to arouse interest from the industry. However, there are certain companies that managed to obtain basil seed oil using a cold pressing method for cosmetic applications; however, this was not to a food grade standard.

### 7.2. Others Uses of Basil Seeds

According to Thessrimuang and Prachayawarakorn [95] and Khazaei et al. [96], the mucilage of basil seeds has an excellent tensile and deformation capacity at maximum loads; thus, it can be used as a biodegradable film and in active packaging for various food applications.

A study by Mezeyová et al. [69] supported the use of basil seeds as a secondary reservoir of selenium due to their ability to absorb this mineral after incorporating it during cultivation. In addition, this seed has the capacity to adsorb several metals, such as copper, cesium, and strontium, in quantities of 400, 160, and 247 mg per g of dry seed, respectively, in contaminated water, making it possible to use them as a sustainable option to bioremediate-contaminated water [28,97,98].

The presence of heavy metals in the ground can affect basil morphology, biomass, and oil content. Moreover, there are several authors that described the capacity of the basil plant to absorb heavy metals from the ground and transport them to the roots, leaves, and flowers; however, there is no information about the presence of heavy metals in basil seeds [99,100,101,102]. In this context, it would be interesting to investigate the heavy metals contents of the seeds in different geographical locations in the future.

## 8. Conclusions

Basil seeds are a source of vegetable compounds, including proteins, omega 3 fatty acids, dietary fiber, minerals, flavonoids, and polyphenols, all of which are attractive characteristics for the food industry and consumers looking for foods with healthy properties. In addition, they have remarkable properties that are beneficial in relation to health and disease prevention. 

Traditionally, basil seeds are included in certain foods and meals in the East; however, in other regions, such as Europe and America, the seeds and their by-products are only beginning to be considered as a functional food. For this reason, more research on basil seeds, their potential health benefits, and their uses in foods is required to enhance the potential of this seed. Future studies could include the cultivation and characterization of basil seeds and their by-products in Latin and North America and their potential use in foods as a functional and/or nutraceutical ingredient.

## Figures and Tables

**Table 1:** The physical properties of basil seeds.

Origin	Length (mm)	Width (mm)	Thickness (mm)	Species	Reference
Iran	3.11	1.82	1.34	*O. basilicum*	[31]
Iran	3.22	1.84	1.37	*O. basilicum*	[32]
Serbia	2.31–2.64	1.30–1.54	0.99–1.14	*O. basilicum*	[29]
India	1.97	1.06	ND	*O. basilicum*	[10]

ND: Not determined.

**Table 2:** The nutritional composition of basil seeds (g/100 g dry weight basis).

References		[10]	[35]	[27]	[6]	[7]	[36]	[34]	[5]	[30]
Component		India	India	Iran	Iran	Iran	Pakistan	Pakistan	Romania	Various Countries **
	Origin									
Moisture		9.6	9.4	5.02–6.24	4.0	ND	5.2	9.2	7.0	5.9–7.8
Protein		14.8	10	17.9–20.16	20.4	22.5	11.4	17.3	15.4	ND
Lipid		13.8	33.0	22.0–24.5	16.6	ND	20.2	9.7	29.0	9.5–19.6
Ash		7.7	5.6	4.7–5.5	8.9	5.11	6.3	5.8	6.5	ND
Carbohydrate		63.8	43.9	47.2–50.1	40.1 *	ND	56.9 *	58 *	47.0	ND
Fiber		22.6	ND	ND	26.2	ND	ND	7.11	ND	ND

ND: not determined. * Determined by difference. ** Singapore, India, Vietnam and Pakistan.

**Table 3:** The amino acid composition of basil seeds (mg/100 mg).

Reference	[45]	[46]
Amino Acids	Hoary Basil (*O. basilicum*)	Holy Basil (*O. tenuiflorum*)
Aspartic acid	4.61	1.45
Serine	3.58	1.00
Glutamic acid	10.55	3.16
Glycine	3.12	0.89
**Histidine**	1.70	0.65
**Arginine**	8.48	2.05
**Threonine**	**2.16**	**0.60**
Alanine	2.65	0.80
Proline	2.25	0.66
Tyrosine	2.08	0.52
**Valine**	2.63	0.77
**Lysine**	1.56	0.54
**Isoleucine**	1.91	0.54
**Leucine**	4.02	1.13
**Phenylalanine**	3.49	0.93
Cysteic acid	ND	0.58
**Methionine sulfone**	ND	0.90
**Tryptophan**	ND	0.96

In bold: essential amino acids. ND: not determined.

**Table 4:** Fatty acid composition (g/100 g) of basil seeds.

References		[51]	[52]	[53]	[47]	[33]	[30]	[42]
Fatty Acids		Canada	Various Countries *	India	Iran	Iran	Various Countries **	Sudan
	Origin							
Palmitic acid (C16:0)		6.8–8.8	5–13	8.0–9.2	4.9	6.23–10.16	5.6–7.7	13.38
Stearic acid (C18:0)		2.0–2.8	2–3	3.6–3.8	2.5	2.97–4.9	2.2–4.4	6.6
Oleic acid (C18:19)		8.7–11.6	6–10	10.3–12.3	7.55	6.2–19.9	5.6–19.4	4.0
Linoleic acid (C18:2n6c)		18.3–21.7	12–32	23.6–26	20.2	16.7–24.9	18.6–85.6	32.2
Linolenic acid (C18:3n3)		57.4–62.5	49–75	49.3–52.4	63.8	42.4–61.9	0.3–66.0	44.0

* Sudan, Germany, and United Arab Emirates. ** Singapore, India, Vietnam, and Pakistan.

**Table 5:** The mineral composition of basil seeds (mg/100 g).

Reference	[34]	[46]
Minerals	*Ocimum basilicum*	*Ocimum tenuiflorum*
Fe	2.27	8.73
Zn	1.58	5.52
Mg	31.55	293.0
Mn	1.01	1.95
K	ND	481.0
Na	ND	2.01
Ca	ND	636.0

ND: not determined.

**Table 6:** The antioxidant activity and polyphenol content in basil seed extracts.

Basil Species Variety	Origin	Solvent (Extraction)	Method AOA	Total AOA		Method TPC	TPC (µg GA/g)	References
				%	(mmol Trolox/Kg)			
*O. tenuiflorum* “Tulsi”	Slovakia	methanol	DPPH	968.49	26.67	Folin–Ciocalteu	1506.55	[69]
*O. basilicum* “Cinamonette”	Slovakia	methanol	DPPH	850.49	26.97	Folin–Ciocalteu	1567.60	[69]
*O. basilicum* “Dark Green”	Slovakia	methanol	DPPH	869.09	26.26	Folin–Ciocalteu	1681.75	[69]
*O. basilicum* L.	Oman	methanol	-	-	-	Folin–Ciocalteu	7857.6	[67]
*O. basilicum* L.	Iran	acetone	ABTS		10.8–35.7	Folin–Ciocalteu	22,900–65,500	[68]
*O. basilicum* L.	Pakistan	ethanol	-	-	-	Folin–Ciocalteu	63,780	[34]
*O. basilicum* L.	Pakistan	n-hexane	DPPH	57.35	-	Folin–Ciocalteu	4890	[36]
*O. basilicum* L	Pakistan	methanol	DPPH	84.59	-	Folin–Ciocalteu	5670	[36]
*O. basilicum* L.	India	petroleum ether	DPPH	73.85	-	-	-	[13]
*O. basilicum* L.	India	methanol	DPPH	34.20	-	-	-	[13]

GA: Gallic acid, standard unit for phenolic content determination. The results are expressed in dry weight. AOA: Antioxidant Capacity Analysis. TPC: Total Phenolic Content. DPPH: (2,2-diphenyl-1-picryl-hydrazyl-hydrate) ABTS: 2 2’-azino-bis(3-ethylbenzothiazoline-6-sulfonic acid).

**Table 7:** Summary of the biological activity of basil seeds and their constituents.

Component/Constituents		Biological Activity	Type of Study	Doses	Results	Reference
Fixed oil (Petroleum ether extract of basil seeds)	α-linolenic acid fatty acids	Anti-inflammatory	Models of carrageenan, leukotriene, and arachidonic acid-induced paw edema in rats.	1.0, 2.0, and 3.0 mL/kg of fixed oil	Significant inhibition of paw edema with 3.0 mL/kg dose. Higher α-linolenic acid content produced a greater inhibition of paw edema.	[75]
		Anti-asthmatic	Histamine-induced bronchospasm in guinea pigs.	0.2 mL and 0.5 mL/kg of fixed oil	Maximum activity observed at 0.5 mL/kg dose of fixed oil for histamine- and acetylcholine-induced bronchospasm.	[76]
			Acetylcholine-induced bronchospasm in guinea pigs.	0.5 mL/kg of fixed oil		
		Anti-inflammatory	Induction of paw edema in rats, viz. carrageenan, serotonin, histamine and prostaglandins (PGE_2_).	0.1 mL/100 g of fixed oil	Fixed oil inhibited hind paw edema induced in rats by treatment with carrageenan, serotonin, histamine, and PGE_2._	
		Antipyretic	Testing it against typhoid-paratyphoid fever A/B vaccine induced pyrexia in rats.	1.0, 2.0, and 3.0 mL/kg of fixed oil	At doses of 1.0 mL/kg or higher, the oil exhibited a defined antipyretic property. The activity at a dose of 3.0 mL/kg was similar to that of aspirin.	[77]
		Analgesic	Methods of tail flapping, tail clip, tail dip, and twisting induced by Acetic acid.	1.0, 2.0, and 3.0 mL/kg of fixed oil	Using an acetic acid-induced writhing method, the oil showed significant inhibition in a dose-dependent manner suggesting its possible mechanism related to the peripheral system.	[78]
		Anti-arthritics	Induction, by injecting a *Mycobacterium tuberculosis* suspension and by injecting a formaldehyde solution into rats.	1.0, 2.0, and 3.0 mL/kg of fixed oil	The fixed oil presented greater anti-arthritis activity at a dose of 3.0 mL/kg, which was similar to the effect of aspirin.	[79]
		Antiulcer	Aspirin-, indomethacin-, alcohol-, histamine-, reserpine-, serotonin-, and stress-induced ulceration in rats and guinea pigs.	1.0, 2.0, and 3.0 mL/kg of fixed oil	The fixed oil possesses greater antiulcer activity at a dose of 3.0 mL/kg	[80]
		Antihyperlipidemic and antioxidant	Application of a diet together with fixed oil and cholesterol in rabbits.	0.8 g/kg of fixed oil	The fixed oil presented a hypocholesterolaemic effect when it was added to the diet for five weeks.	[81]
		Antimicrobial	Determination by paper disc diffusion method.		Fixed oil has good antibacterial activity against *S. aureus*, *B. pumilus* and *P. aeruginosa*, where *S. aureus* was the most sensitive organism (zone of inhibition 0.8 mm).	[83]
		Anticoagulant	Intraperitoneal application of fixed oil to rats.	3.0 mL/kg of fixed oil	Fixed oil increased the blood-clotting time and the percentage increase was comparable to aspirin.	[82]
		Anticancer	20-Methylcholanthrene-induced fibrosarcoma tumors injected subcutaneously in the thigh region of mice.	100 mL/kg of fixed oil	The fixed oil presented chemopreventive efficacy at a dose of 100 mL/kg, which was comparable to the effect of Vitamin E	[83]
Phytochemical (petroleum ether extract of basil seeds)	Alkaloids, flavonoids, carbohydrates, tannins, terpenoids	Antioxidant	DPPH radical scavenging assay		73.85% of the antioxidant capacity of *O. basilicum* seeds results from the contribution of phenolic compounds.	[13]
		Anticancer	MTT (3-[4,5-dimethylthiazol-2-yl]2,5-diphenyl tetrazolium Bromide) assay.		The cell viability percentage showed a maximum activity at a lower concentration, i.e., 12.5 μg/mL.	
		Antimicrobial	Determination by using the well diffusion method.		Highest zone of inhibition was observed at 100 mg/mL concentration against *P. aeruginosa.*	
Water soluble polysaccharides (aqueous extracts)	Glucose, galacturonic acid, rhamnose, mannose, arabinose, glucuronic acid, and galactose	Antidiabetic	Measuring the inhibitory activity for protein tyrosine phosphatase 1B in vitro.		Inhibitory activity for protein tyrosine phosphatase 1B IC50 = 8.20 µg/mL	[14]
Peptides (Hydrolyzed and non-hydrolyzed extracts)	P1 (ACGNLPRMC) P2 (ACNLPRMC) P3 (AGCGCEAMFAGA)	Antioxidant activity α-amylase inhibitory activity.	DPPH and FRAP methodPotential α-amylases inhibitor peptides		Peptides can be used as therapeutic agents to reduce the risk of oxidative stress and to prevent type-2 diabetes.	[72]

IC50: corresponds to the half maximal inhibitory concentration; FRAP: Ferric reducing antioxidant power.
